# Splenic infarction and infectious diseases in Korea

**DOI:** 10.1186/s12879-020-05645-9

**Published:** 2020-12-02

**Authors:** Jae Hyoung Im, Moon-Hyun Chung, Hye-Jin Lee, Hea Yoon Kwon, Ji Hyeon Baek, Ji-Hun Jang, Jin-Soo Lee

**Affiliations:** 1 Division of Infectious Diseases, Department of Internal Medicine, Inha University School of Medicine, Incheon, 22212 Republic of Korea; 2 Department of Internal Medicine, Seogwipo Medical Center, Jeju-do, Republic of Korea; 3 Translation Research Center, Inha University School of Medicine, Incheon, Republic of Korea; 4 Department of Hospital Medicine, Inha University School of Medicine, Incheon, 22212 Republic of Korea

**Keywords:** Etiology, Infection, Infectious endocarditis, Splenic infarction, Intracellular organisms

## Abstract

**Background:**

The spleen contains immune cells and exhibits a pattern of infarction different from other organs; as such, splenic infarction (SI) may provide important clues to infection. However, the nature of the relationship between SI and infectious disease(s) is not well understood. Accordingly, this retrospective study investigated the relationship between SI and infection.

**Methods:**

Hospital records of patients with SI, who visited Inha University Hospital (Incheon, Republic of Korea) between January 2008 and December 2018, were reviewed. Patient data regarding clinical presentation, causative pathogens, risk factors, and radiological findings were collected and analyzed.

**Results:**

Of 353 patients with SI, 101 with infectious conditions were enrolled in this study, and their data were analyzed to identify associations between SI and infection. Ten patients were diagnosed with infective endocarditis (IE), and 26 exhibited bacteremia without IE. Twenty-seven patients experienced systemic infection due to miscellaneous causes (negative result on conventional automated blood culture), including the following intracellular organisms: parasites (malaria [*n* = 12], babesiosis [n = 1]); bacteria (scrub typhus [*n* = 5]); viruses (Epstein–Barr [n = 1], cytomegalovirus [n = 1]); and unidentified pathogen[s] (*n* = 7). Splenomegaly was more common among patients with miscellaneous systemic infection; infarction involving other organs was rare. Thirty-eight patients had localized infections (e.g., respiratory, intra-abdominal, or skin and soft tissue infection), and most (35 of 38) had other risk factors for SI.

**Conclusions:**

In this study, various infectious conditions were found to be associated with SI, and intracellular organisms were the most common causative pathogens. Further studies are needed to examine other possible etiologies and the underlying pathophysiological mechanisms.

## Background

Splenic infarction (SI) is generally asymptomatic; however, it may occasionally result in severe complications including bleeding, rupture, pseudocyst formation, and death [1]. SI is mainly caused by thrombosis or vascular injury [2] mediated by arrhythmia, cancer, liver cirrhosis, pancreatitis, trauma, vascular procedures, infective endocarditis (IE), and/or coagulopathy [3].

In clinical practice, SI is often detected incidentally when abdominal *computed tomography* (CT) is performed to identify the cause(s) of fever. While several investigations have shown that various pathogens can cause SI [4–8], comprehensive studies investigating the association between SI and infection are limited. One retrospective study reported that 4 of 32 patients with SI had infections [3], whereas another identified infection as the causative factor in 11 of 89 patients with SI [9]. Based on the results of previous studies, assessing the etiology of SI has the following limitations: a lack of extensive information regarding the relationship between SI and infection; and a lack of a sufficient number of cases to enable in-depth analysis.

Because the spleen contains immune cells, it exhibits a pattern of infarction different from that of other organs. In addition, these other aspects can be an important clue to the diagnosis of infectious diseases. However, the relationship with infection is often overlooked. Lack of understanding these relationships can lead to unnecessary and/or time-consuming tests and treatments or misdiagnosis. In addition, inappropriate examination or use of empirical antibiotics can occur. Therefore, to bridge this gap in knowledge, we investigated the relationship between SI and infection by conducting a retrospective analysis of the clinical and demographic data of patients with SI and coexisting infections.

## Methods

### Overall design and study population

This study was designed as a retrospective investigation of patients who were diagnosed with both SI and infectious disease at Inha University Hospital (Incheon, Republic of Korea) between January 2008 and December 2018. Data regarding patient demographic and clinical characteristics were collected from medical records. The inclusion criteria for this study were a diagnosis of SI and evidence of pathogenic infection. Because the differentiation between SI due to various metastatic cancers is limited, patients with solid organ malignancy were excluded from this study. Patient medical records were reviewed for risk factors for SI including trauma, vasculitis, pancreatitis, pancreatic tumor, surgical technique, invasive procedures, hematological malignancy, liver cirrhosis with portal hypertension, atrial fibrillation, atherosclerotic disease, and hypercoagulative status. SI-associated radiological findings were analyzed in the context of splenomegaly with signs of obstruction/infarction in other organs. Patients with liver cirrhosis and/or hematological malignancy were excluded from the subgroup analysis of patients with splenomegaly because splenomegaly can occur as a result of these conditions.

### Case definition

Diagnosis of SI was based on ≥1 of the following CT findings: peripheral wedge-shaped lesion with low attenuation; multiple heterogeneous lesions with patchy enhancement; or lesions with extensive or complete low attenuation [10].

Evidence supporting the presence of infectious disease was as follows: fever, with temperature > 37.7 °C; increased levels of inflammatory markers (C-reactive protein > 0.05 mg/dL or leukocyte count > 10,000/μL); and/or general clinical signs and symptoms of infection. The diagnosis of infection was confirmed using blood culture studies, polymerase chain reaction techniques, serological examinations, microscopic examinations, and histopathological studies. IE was defined according to the Duke criteria [11]. Bacteremia was defined as a positive result on blood analysis using an automated blood culture system (BACTEC, BD Biosciences, Franklin Lakes, NJ, USA). Miscellaneous systemic infection was defined as systemic infection in the absence of IE or bacteremia, assessed using a conventional automated blood culture system. Localized infections were defined by the presence of typical features indicative of the involvement of a single organ system observed during physical examination, laboratory tests, or radiological examination at the time of admission. Respiratory, urinary, or gastrointestinal tract infections, skin and soft tissue infections, and intra-abdominal, hepatobiliary, or central nervous system infections are some examples of localized infections involving a single organ system.

### Data analyses

The Fisher’s exact test was used to compare risk factors and radiological findings of SI. All data analyses were performed using SPSS version 18 (IBM Corporation, Armonk, NY, USA) and differences with *P* < 0.05 were considered to be statistically significant.

### Ethics statement

Ethics approval was obtained from the Institutional Review Board of Inha University Hospital (Institutional Review Board IRB No.: 2019–06-009). All patient data were anonymized; as such, the need for consent for publication was waived.

## Results

### Demographic characteristics of patients

Among 353 patients with SI, 252 with no evidence of infection or a history of solid cancer(s) were excluded from the study. Data from the remaining 101 patients were analyzed to assess the relationship between infection and SI. The mean (± standard deviation) age of the patients was 59.1 ± 17.3 years, and 49.5% of the cohort was female. Thirty-nine patients reported abdominal pain, and 25 died: 1 due to splenic rupture, 8 from sepsis; 2 of cardiogenic events; 5 from acute respiratory distress syndrome, 3 from hypovolemic shock; and 1 from liver failure. Splenic rupture was suspected in 3 patients, 1 of whom died.

### Causes of infection with SI

The etiologies of infection in the study population are summarized in Table 1. Ten (9.9%) patients had IE, 26 (25.7%) had bacteremia without IE, 27 (26.7%) had miscellaneous systemic infection, and 38 (37.6%) had a localized infection. The most common isolated bacterial pathogens were as follows: *Staphylococcus aureus* (*n* = 7 [6.9%]), *Escherichia coli* (*n* = 6 [5.9%]), viridans group *Streptococcus* (*n* = 3 [3.0%]), and *Enterococcus faecalis* (n = 3 [3.0%]).

**Table 1:** Causes of infections in patients with splenic infarction

Variables	Number of patients	Detailed etiology
**Systemic infection (*N*** **= 63)**		
Infective endocarditis	10	1 *Escherichia faecalis*, 1 *Enterococcus gallinarium* 1 *Staphylococcus aureus*, 1 Viridans group *Streptococcus*, 6 Culture-negative
Bateremia without infective endocarditis	26	6 *S. aureus*, 6 *E. coli*, 2 Viridans group *Streptococcus*, 2 *Klebsiella pneumoniae*, 2 *Enterococcus faecalis*, 2 *Acinetobacter baumanii*, 1 *E. gallinarium*, 1 cogulase-negative *Staphylococcus*, 1 *Comamonas testosteroni*, 1 *Candida albicans*, 1 unidentified anaerobe
Miscellanous	27	
*Malaria*	12	12 *Plasmodium vivax*
*Tsutsugamushial disease*	5	
*Babesia*	1	1 *Babesia microti*
*Epstein Barr virus*	1	
*Cytomegalovirus*	1	
*Other, not identified*	7	1 Suspected *Mycoplasma* infection
**Localized infection (*N*** **= 38)**		
*Respiratory tract infection*	11	9 Pneumonia (1 *Mycobacteriium tuberculosis*), 1 Empyema, 1 Pharyngitis
*Skin and soft tissue infection*	8	4 Cellulitis, 2 Psoas abscess, 1 Necrotizing fasciitis, 1 Prosthetic infection
*Hepatobilliary infection*	8	5 Cholecystitis, 3 Liver abscess (2 Pyelophlebitis)
*Intra-abdominal infection*	9	7 Peritonitis, 2 Enteritis
*Urinary tract infection*	2	2 Pyelonephritis
Total	101	

Of patients with miscellaneous systemic infection, *Plasmodium vivax* was the most commonly isolated pathogen (*n* = 12), followed by *Orientia tsutsugamushi* (*n* = 5), *Babesia microti* (n = 1), cytomegalovirus (n = 1]), and Epstein–Barr virus (n = 1). Causative pathogens could not be identified in the remaining 7 patients.

In the 38 patients with a localized infection, the most common clinical presentation was respiratory infection (*n* = 11), followed by intra-abdominal infection (*n* = 9), hepatobiliary infection (*n* = 8), skin and soft tissue infection (n = 8), and urinary tract infection (*n* = 2).

### Risk factors for SI in patients with infection

Among the 101 patients, 63 had ≥1 risk factors for SI, whereas 38 did not have any risk factors. Among patients with risk factors, 27 had a condition that could directly affect the pancreatic vessel(s) (hematological malignancy [*n* = 12], pancreatitis/pancreatic tumor [*n* = 8], connective tissue diseases [*n* = 3], portal vein thrombosis [n = 3], and history of trauma [n = 1]). Of the remaining 36 patients (excluding conditions capable of directly affecting the splenic artery), the most common comorbidities were atherosclerotic disease(s) (*n* = 22), followed by atrial fibrillation (*n* = 13), liver cirrhosis (*n* = 8), and a hypercoagulative status when including duplication (n = 8) (Table 2). Among patients without any risk factors, miscellaneous systemic infection was most commonly observed (*n* = 21).

**Table 2:** Risk factors of splenic infarction

	Systemic infection			Localized infection	Total
	Infective endocarditis	Bacteremia without infective endocarditis	Miscellaneous		
**Without any risk (N = 38)**	6	8	21	3	38
**With risk factors (N = 63)**	4	16	6	37	63
Trauma/surgery (N = 1)				1	1
Pancreatitis/Pancreatic tumor (N = 8)		3		5	8
Hematologic malignancy (*N* = 12)	2	4		6	12
Connective tissue diseases (N = 3)				3	3
Portal vein thrombosis (*N* = 3)		2		1	3
Atrial fibrillation (N = 13)					
*without other risk factors*		1	1	1	3
*with atherosclerotic diseases*		1	2	5	8
*with atherosclerosis and hypercoagulative status*				1	1
*with liver cirrhosis*				1	1
Atherosclerotic Diseases (N = 22)					
*without other risk factors*	2	4	3	2	11
*with atrial fibrillation*	Same as atrial fibrillation + atherosclerotic diseases				
*with atrial fibrillation and hypercoagulative status*	Same as atrial fibrillation + atherosclerotic Diseases + hypercoagulative status				
*with hypercoagulative status*		1			1
*with liver cirrhosis*				1	1
Hypercoagulative status (N = 8)					
*without other risk factors*		1		4	5
*with atherosclerotic diseases*	Same as atherosclerotic diseases + hypercoagulative status				
*with atherosclerotic diseases and atrial fibrillation*	Same as atherosclerotic diseases + atrial fibrillation + hypercoagulative status				
Liver cirrhosis (N = 8)					
*without other risk factors*		1		4	5
*with atherosclerotic diseases*	Same as atherosclerotic diseases + liver cirrhosis				
*with atrial fibrillation*	Same as atrial fibrillation + liver cirrhosis				
Total	10	24	27	40	101

Of the 38 patients with localized infections, 35 had ≥1 risk factors. This is probably because a localized infection alone is unlikely to cause SI. Three of the patients with localized infections developed SI but did not exhibit any risk factors. One patient had necrotizing fasciitis due to *Streptococcal pyogenes*, the second had liver abscess due to *Klebsiella pneumoniae*, and the third had cellulitis with no proven pathogenic cause (a patient with end-stage renal disease).

### Radiological findings of SI in patients with infection

Several differences in the type of infection, depending on the presence or absence of splenomegaly, were found. After excluding patients with liver cirrhosis and/or hematological malignancy, 77 patients were included in the subgroup analysis for assessment of features associated with splenomegaly. Fifty-nine patients without splenomegaly had various types of infections: IE (*n* = 6); bacteremia without IE (*n* = 20); miscellaneous systemic infections (*n* = 12); and localized infections (*n* = 21). However, most patients with splenomegaly (15 of 18 [83.3%]) had miscellaneous systemic infections. Only 3 patients had splenomegaly (2 with culture-negative IE and 1 with both urinary tract infection and polycythemia vera) in the non-miscellaneous systemic infections group (Table 3).

**Table 3:** Radiologic findings in patients with splenic infarction

		Systemic infection			Localized infection	Total	χ2	*P*-value
		Infective endocarditis	Bacteremia without infective endocarditis	Miscellaneous				
Splenomegaly (*N* = 77)	No (Normal size spleen)	6	20	12	21	59	16.797	< 0.001
	Yes (Splenomegaly)	2	0	15	1	18		
	Total	8	20	27	22	77		
Mulitple site infarction (*N* = 101)	No (Only splenic)	6	22	25	31	84	3.659	0.067
	Yes (Multiple organ)	4	4	2	7	17		
	Total	10	26	27	38	101		

SI caused by miscellaneous infections tended to involve only the spleen rather than multiple organs. However, there was no statistically significant difference in the incidence of miscellaneous infections between patients with SI alone and those with multiple organ infarction/obstruction.

## Discussion

SI was found in 101 of 353 patients in this study. This is a fairly high rate considering the relationship between splenic infarction and infection, which has been of low interest to physicians. In some patients in this study, the infection may simply be a secondary infection. However, we deliberately included these blood vessel compromised statuses. This is because the purpose of this study was to assume the situation when a clinician encounters a patient with splenic infarction and infection, and to inform about its management. To this end, we investigated various etiologies to determine the overall relationship between infection and SI and found that the etiologies were largely divided into the following groups: thrombotic events due to IE or sepsis; events due to the presence of an intracellular organism; and events due to the synergy of a localized infection and other risks.

Thrombogenic events can be considered the main mechanism underlying infection-induced SI (35.3% in this study). Infection can cause thrombosis through various mechanisms. In the presence of inflammatory conditions, increased cytokine production due to sepsis disrupts the coagulation system [12], activating platelets by the action of pro-inflammatory mediators, such as platelet-activating factors [13], as well as the action of P-selectin, which increases systemic inflammation and leads to platelet adhesion [14]. The functioning of the elements of the anti-coagulation mechanism, including anti-thrombin, the protein C system, and inhibitors of the tissue factor pathway, can be compromised by infection [15]. In infectious endocarditis, the common pathogens are *Staphylococcus*, *Streptococcus*, and *Enterococcus*, as well as other fungal species. There may be differences in the degree to which IE is triggered even within a single species. For example, serotypes 2, 5, and 8 of *S. aureus* are more dangerous [16–18] in thrombotic events. Decreased expression of fibronectin-binding proteins (FnBPA and FnBPB) produces less IE [19], and loss of expression of GspB in *Streptococcus* is indicated by reduced toxicity of IE [20, 21]. On the other hand, *Escherichia* and *Klebsiella*, which are common causes of bacteraemia, have relatively less IE unlike *Staphylococcu*s and the viridans group of *Streptococcus* [22]. This is because the species have a low ability to attach to a non-bacterial thrombotic embolism. In this study, *Escherichia* or *Klebsiella* were found in bacteraemia without IE. In an animal model, administration of lipopolysaccharide purified from Escherichia, Klebsiella, and Salmonella induced a septic state. Host-derived responses to this cell wall component are important in inducing a pro-thrombotic state. The binding of LPS with monocytes/macrophages, platelets, and endothelial cells activates coagulase factors and pro-inflammatory responses, resulting in a pro-coagulant state [23]. Moreover, these pro-inflammatory molecules reduce the levels of anti-coagulant proteins, which can be related with DIC [24, 25]. The occurrence of antiphospholipid syndrome or disseminated intravascular coagulation in association with severe infection can also be considered a thrombogenic event.

In this study, 27 (26.7%) patients were classified with miscellaneous systemic infections, with intracellular organisms representing the causative pathogens in most of these cases. Malaria and babesiosis have previously been reported to cause SI [26, 27]. Both are parasitic diseases in which the parasites infect red blood cells (RBCs). In these diseases, parasitemia and the destruction of RBCs result in hemostasis and cytokine-induced thrombosis, which serve as the main causes of SI; however, the exact mechanism by which SI occurs in these diseases remains unclear. *Orientia tsutsugamushi* infection was frequently identified in several of our patients. This organism is known to cause endothelial dysfunction, leading to the triggering of fibrin formation and platelet adhesion and aggregation by endothelial cells [14]. Additionally, endothelial dysfunction contributes to the impairment of the protein C system [14]. Antiphospholipid syndrome is another cause of endothelial dysfunction [28]. In addition to the conditions revealed in this study, Q fever, herpes infection, brucellosis, typhoid, and murine typhus [29] are others that cause SI due to endothelial dysfunction. When physicians encounter patients with SI, they must consider the possibility of infection with different pathogens capable of causing endothelial dysfunction. It noteworthy that most of the patients in this group (miscellaneous systemic infection) also had splenomegaly. In addition to endothelial dysfunction, vessel compression accompanying splenomegaly may be considered one of the causes of splenomegaly; however, no studies have clearly delineated this. Identifying the presence or absence of splenomegaly in SI patients may be helpful in differentiating those with intracellular organism(s) from those with SI. In addition, the fact that the patients in the miscellaneous systemic infection group did not exhibit multi-organ occlusion can help differentiate them from other SI patient groups.

Most patients with localized infections and SI had at least one risk factor. Risk factors, including trauma, surgery, pancreatitis, pancreatic tumor, and portal vein thrombosis, which were seen at a rate of 11.9% in this study, are generally considered to be unrelated to infection, except for pancreatic abscess, splenic abscess, and pylephlebitis (liver abscess) [30, 31]. Hematological malignancy, vasculitis, hypercoagulative status, and atrial fibrillation, however, are believed to lead to SI because of their varied associations with infection. Hematological malignancy is believed to be associated with splenomegaly and/or hyperviscosity under acute leukemic conditions. However, infections due to an immunocompromised status should always be monitored, and attention should be devoted to the increased tendency of thrombosis due to infections. Patients with connective tissue disorders may develop SI as a result of vasculitis, splenomegaly, and antiphospholipid syndrome (22). Infections in patients with vasculitis are generally believed to be secondary to an immunocompromised status; nevertheless, the possibility of infection exacerbating connective tissue diseases should also be taken into consideration [32]. Exacerbation of atherosclerosis due to infection is sometimes ignored in clinical practice. Our findings revealed that atherosclerotic disease was the most common risk factor (22/101 [21.8%]) for SI in patients with infection. Several studies have shown that infection exacerbates atherosclerosis [33]. However, acute infection alone is unlikely to cause a sufficiently rapid aggravation of atherosclerosis to cause SI. Other studies suggest that occlusion may be associated with vasospasm. Arterial spasms can be caused by the increased production of the cytokine interleukin-1 and reduced bioavailability of nitric oxide during acute infection (19). In patients with atrial fibrillation, infections can promote SI by enhancing thrombogenic tendencies. Infection can lead to atrial fibrillation; however, only 2 of our patients developed paroxysmal atrial fibrillation, and most patients with atrial fibrillation had experienced it previously. If a clinician detects splenic infarction in patients with localized infection, it is necessary to be careful about multiple factors.

These conditions must be differentiated using various approaches. First, it is important to differentiate between bacteremia and IE. Efforts should then be made to identify the underlying disease(s) or risk factors. If no other risk factors are identified, infection by intracellular organisms should be ruled out. In this study, we found that the presence of intracellular organisms was associated with splenomegaly without infarction in other organs. These findings may help differentiate the causes of various types of SI; nevertheless, a comprehensive evaluation of the patient’s underlying disease and history is important. Furthermore, it is difficult to diagnose SI with a localized infection alone, and in this case, the physician must check whether the patient with SI has other risk factors.

Our study had a few limitations, the first of which was its retrospective design; however, a prospective study investigating this topic would be difficult to perform due to the nature of SI. To overcome the drawbacks of a retrospective study design, we considered various risk factors for SI and analyzed their potential correlations with the development of SI. Second, specific pathogen(s) could not be identified in some of the investigated cases. Third, the findings of this study are based on the experience at a single tertiary hospital; as such, it may be difficult to generalize these findings elsewhere, particularly because diseases, such as malaria and scrub typhus, exhibit significant variation in regional incidence. Fourth, the small number of SI patients was insufficient to address all etiologies. We additionally encountered 2 cases of SI due to *Salmonella typhii* and *Candida albicans* after the set study period. It is believed that many more pathogens cause SI. However, the purpose of this study was to examine the nature of the relationship between infection and SI rather than to analyze the exact etiology of SI itself.

## Conclusion

SI was associated with various infections. In addition to sepsis and IE, various intracellular organisms were implicated in the pathogenesis of infection-induced SI. The endothelial dysfunction caused by intracellular organisms may be a mechanism leading to SI; nevertheless, further research investigating this topic is warranted. Because various risk factors are associated with infections, we suggest considering both clinical and radiological findings from SI patients when making a diagnosis.

## Data Availability

The datasets used during the current study are available from the corresponding author upon reasonable request.

## Abbreviations

CT: Computed tomography
IE: Infective endocarditis
RBCs: Red blood cells
SI: Splenic infarction
